# Evidence of different climatic adaptation strategies in humans and non-human primates

**DOI:** 10.1038/s41598-019-47202-8

**Published:** 2019-07-30

**Authors:** L. T. Buck, I. De Groote, Y. Hamada, B. R. Hassett, T. Ito, J. T. Stock

**Affiliations:** 1 PAVE research group, Department of Archaeology, University of Cambridge, Pembroke Street, Cambridge, CB2 3QG UK; 2 Human Origins Research Group, Department of Earth Sciences, Natural History Museum, Cromwell Road, London SW7 5BD UK; 3 Department of Anthropology, University of California Davis, 1 Shields Avenue, Davis, 95616 CA USA; 4 School of Natural Science and Psychology, Liverpool John Moores University, James Parsons Building, Byrom Street, Liverpool, L3 3AF UK; 5 Primate Research Institute, Kyoto University, Inuyama, Aichi 484-8506 Japan; 6 Institute of Archaeology, University College London, 31-4 Gordon Square, London, WC1H 0PY UK; 7 Department of Anthropology, Western University, London, Ontario, N6A 3K7 Canada; 8 Department of Archaeology, Max Planck Institute for the Science of Human History, Kahlaische Strasse 10, D-07745 Jena, Germany

**Keywords:** Evolutionary ecology, Biological anthropology

## Abstract

To understand human evolution it is critical to clarify which adaptations enabled our colonisation of novel ecological niches. For any species climate is a fundamental source of environmental stress during range expansion. Mammalian climatic adaptations include changes in size and shape reflected in skeletal dimensions and humans fit general primate ecogeographic patterns. It remains unclear however, whether there are also comparable amounts of adaptation in humans, which has implications for understanding the relative importance of biological/behavioural mechanisms in human evolution. We compare cranial variation between prehistoric human populations from throughout Japan and ecologically comparable groups of macaques. We compare amounts of intraspecific variation and covariation between cranial shape and ecological variables. Given equal rates and sufficient time for adaptation for both groups, human conservation of non-human primate adaptation should result in comparable variation and patterns of covariation in both species. In fact, we find similar amounts of intraspecific variation in both species, but no covariation between shape and climate in humans, contrasting with strong covariation in macaques. The lack of covariation in humans may suggest a disconnect in climatic adaptation strategies from other primates. We suggest this is due to the importance of human behavioural adaptations, which act as a buffer from climatic stress and were likely key to our evolutionary success.

## Introduction

Understanding the nature of the adaptations that enabled *Homo sapiens* to become such a successful species, colonising the vast majority of ecological niches globally, is a key question in the study of human evolution. Although other hominin taxa, such as *H*. *erectus*, adapted to novel habitats during periods of range expansion^1^, no other hominin has ever inhabited such a diversity of environments and it can be argued that the strategies enabling this expansion are the defining characteristics of *H*. *sapiens*^2,3^.

For any species, climate results in important stressors, which present amongst the greatest challenges in new environments. It has long been appreciated that amongst the suite of climatic adaptations employed by mammals are skeletal changes in size and shape such as those governed by Bergmann’s and Allen’s rules. These rules state that a larger, relatively rounder shape with short appendages is advantageous in conserving heat in cold climates, while the converse applies in hot climates^4–7^. In the primate (including human) cranium these rules seem to be obeyed by selection for larger, rounder neurocrania and flatter, broader faces in cold-adapted forms^8–20^. Internal and external nasal morphology is also a key site of climatic adaptation, changing form to optimise heat and moisture retention/loss depending on the requirements of the habitat^12,17,20–25^ and likely affecting surrounding craniofacial morphology in turn^26,27^.

In a recent paper^12^, we showed that Japanese macaques from different latitudes throughout the Japanese Archipelago show craniofacial and postcranial differences in morphology which correspond with neither dietary differences nor phylogenetic patterns. The morphology characterising northerly macaques relative to southerly macaques resembles the characteristic differences between very high latitude people and those from lower latitudes^16,18,19^. This suggests that humans do not diverge from a non-human primate pattern of adaptation in this respect. Here we seek to build on that finding and begin to investigate whether there is also a comparable amount of cranial adaptation in humans. That is to say, does comparable climatic stress lead to comparable cranial adaptation in humans and in non-human primates, or do similarities between northern Japanese macaques and humans from much higher latitudes imply that more severe cold stress is required to impact the human phenotype? To address this question, we compare differences in craniofacial morphology between groups of Japanese macaques from sites throughout Japan with ecologically comparable groups of Japanese prehistoric foragers (Jomon). Japan is the ideal site for this research due to its great range of climates within a relatively small geographic area^28^ and the presence of humans and a geographically sympatric non-human primate species, Japanese macaques (*Macaca fuscata*), throughout most of this climatic range.

The Jomon were a forager culture spread throughout the Japanese Archipelago from ~15,000 to ~2,000 BP^29^. Despite considerable evidence for morphological homogeneity between the Jomon of different regions^30–32^, there is some evidence for potentially climate-related differences in their craniofacial morphology. Jomon from Hokkaido have been shown to have larger cranial vaults, lower faces and larger orbits compared to Honshu Jomon as a whole^30^, whilst compared to southern Honshu and Kyushu, northern Honshu Jomon (no Hokkaido Jomon were included) have higher, narrower crania^33^. Compared to the cranial data, there is a greater degree of consensus that Jomon postcrania conform to ecological expectations of size, obeying Bergmann’s rule, although limb proportions do not seem to obey Allen’s rule^32,34,35^. This potential difference in responsiveness to climate between the cranium and postcrania may reflect differences in plasticity and canalisation inherent to the different skeletal regions^36,37^. To the best of our knowledge, the current study is the first systematic analysis of Jomon cranial morphology using 3D (three-dimensional) shape data generated using geometric morphometric methods (GMM) to compare ecogeographic patterns throughout Japan. GMM preserve the geometry of shapes under analysis and allow the modelling of shape differences within a sample that are associated with other types of variable, such as ecological data.

Here we use GMM and multivariate statistical analyses to investigate amounts of intraspecific variation and the covariation between cranial shape and ecological variables in Japanese macaques and Jomon foragers. We compare the amount of cranial intraspecific variation within species, the strength of covariation between cranial shape and ecology, the importance of different ecological variables to this covariation, and whether the shape associated with ecological variation fits expectations of climatic adaptation. If humans retain both the pattern and amount of non-human primate climatic adaptation we would expect to see similar amounts of intraspecific variation in cranial shape and similar patterns of covariation between cranial shape and ecology within both groups. Greater intraspecific variation and stronger covariation between cranial shape and ecology in the Jomon would be consistent with an enhanced skeletal adaptability, which has been argued to be amongst the adaptive suite that enabled successful global colonisation in humans^1,2^. Conversely, less cranial intraspecific variation and weak/absent covariation between shape and ecology in humans, could suggest reduced skeletal adaptation compared to macaques. This scenario would be consistent with the argument that physical adaptation may be less important than behavioural mechanisms in human adaptation to ocal ecology^38,39^. The latter would underline the importance of human behavioural adaptations such as fire, subsistence technology, clothing, shelter and flexible, social behaviour during the colonisation of novel habitats in our evolutionary history^3,40^. Whilst humans undoubtably adapt both physically and behaviourally to their environments, a difference between the human and non-human primate adaptive responses to climate could suggest that the human adaptive suite is a predominantly behavioural one, differentiating us from our closest relatives.

## Results

### Within-group variation in macaques and Jomon

Mean Procrustes distances and their standard deviations show that the amount of intraspecific variation is very similar for both macaque and Jomon samples (Table 1). For neither macaques nor Jomon is any one group or individual consistently far from the mean shape, showing that no one group or individual drives the amount of variation.

**Table 1:** Procrustes distances within samples for all three analyses.

	Macaques			Jomon		
Analysis	Mean	Range	SD	Mean	Range	SD
CF	0.07	0.05 (Shima) to 0.09 (Shimo)	0.01	0.07	0.05 (Taka) to 0.11 (Ebi)	0.01
F	0.07	0.04 (Shima) to 0.12 (Shimo)	0.02	0.09	0.06 (Kita) to 0.14 (Yoshi)	0.02
NC	0.07	0.04 (Yaku) to 0.13 (Shima)	0.02	0.06	0.03 (KO) to 0.12 (Taka)	0.02

CF: craniofacial landmark set, F: facial landmark set, NC: neurocranial landmark set. Mean Procrustes distances from individuals to mean shapes, greatest and smallest Procrustes distances from an individual to the mean shape (range), and standard deviations of mean distances (SD) are given for macaques and Jomon for each analysis. Sites in brackets, Shima: Shimane, Shimo: Shimokita, Yaku: Yakushima, Taka: Takasago, Ebi: Ebishima, Kita: Kitakogane, Yoshi: Yoshigo, KO: Kotan Onsen, see Supplemental Information section S1.1 for more sample details.

### Covariation between craniofacial shape and ecology

#### Macaques

**Figure 1:**
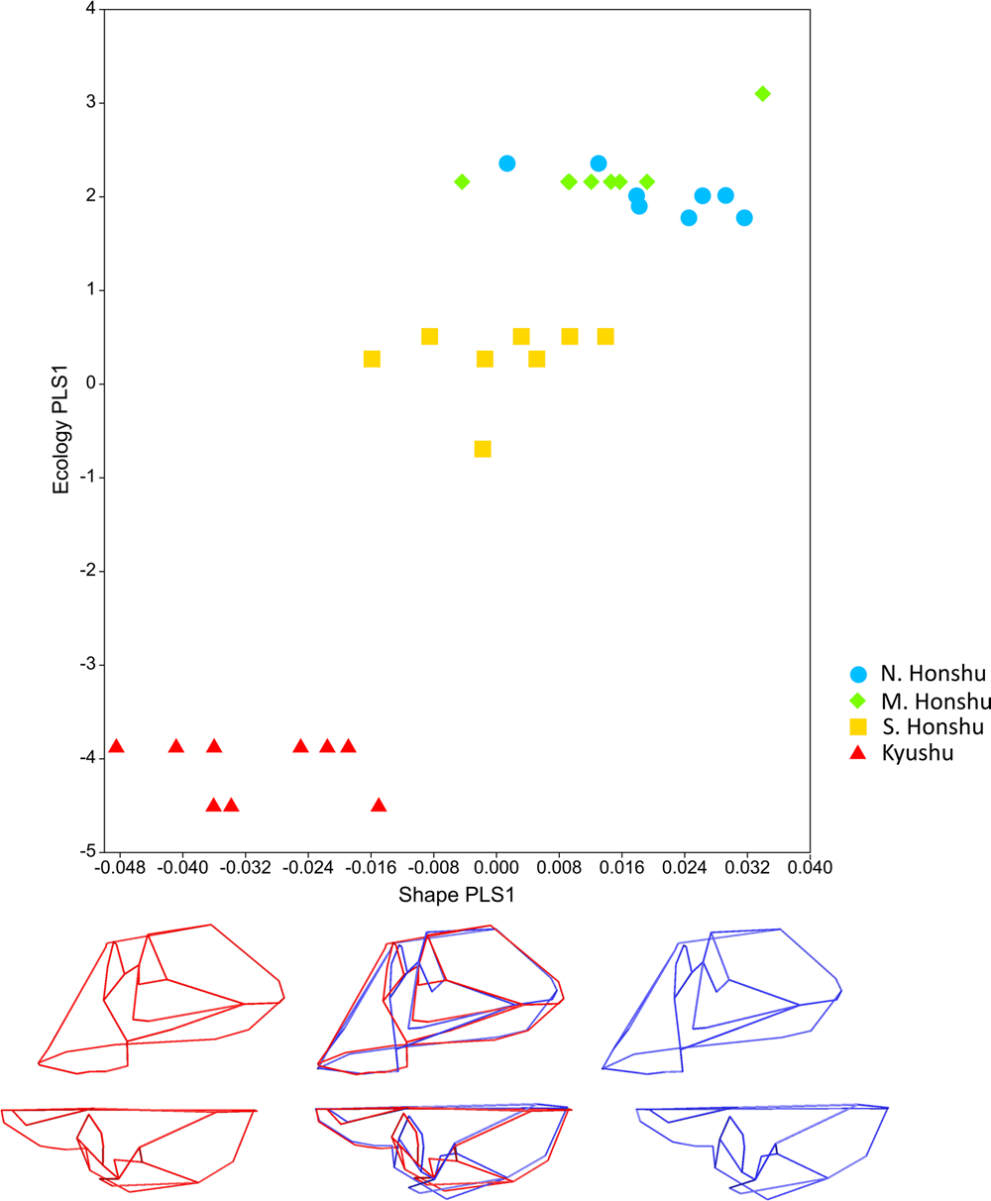
Results from 2-B PLS craniofacial analysis. Block 1 (shape) PLS1 scores against block 2 (ecology) PLS1 scores in craniofacial analysis of macaques (reduced sample). Points coloured by latitude group: light blue circles: N. Honshu, green diamonds: M. Honshu, yellow squares: S. Honshu, red triangles: Kyushu. Mean shape warped to show variation along shape PLS (−0.05 to 0.04 Procrustes distance) (minimum: red, maximum: blue) from side (top), and top (bottom) views. Wireframes as shown in Fig. 6.

Partial least squares (PLS) analysis of craniofacial shape and ecology in the macaque reduced sample (n = 33, see Methods) shows a moderately strong, significant association between blocks (RV coefficient: 0.42, p < 0.001). There are also strong, significant singular values and pairwise comparisons for PLS1 (93.72% total covariation between blocks): singular value = 0.05, p < 0.0001; correlation = 0.89, p < 0.001. The two most northerly groups of macaques largely overlap on this PLS but there is differentiation between them and both southern Honshu and Kyushu macaques (Fig. 1).

All the climatic variables (Table S1.2.4) load PLS1 fairly evenly (mean temperature = −0.39 to temperature range = 0.36), with altitude a much less important influence (0.17). These loadings show that more northerly individuals, experiencing lower temperatures/levels of precipitation and greater temperature ranges, score higher on the shape factor of PLS1 (Fig. 1). Higher scoring individuals have broader faces, particularly in the cheek region, with more anteriorly placed zygomatics. They have narrower noses and more retracted upper faces. The neurocrania of higher scoring individuals are superior-inferiorly shorter and medio-laterally narrower, but the greatest difference is in their relatively shorter anterior-posterior dimensions, which make the neurocrania overall more globular (Fig. 1). This variation in shape is significantly different between latitude groups; there are significant differences in block 1 PLS1 scores between all groups except mid Honshu and both north and south Honshu (SI Section S2.1, Table S2.1.2).

#### Jomon

There is no significant association between Jomon PLS blocks of ecology and craniofacial shape data (RV coefficient: 0.17, p > 0.05), nor do any of the individual pairs of PLS dimensions reach significance (p values all >0.05) (Tables S2.2.1 and 2.2.2, Fig. S2.2.1).

### Covariation between facial shape and ecology

#### Macaques

**Figure 2:**
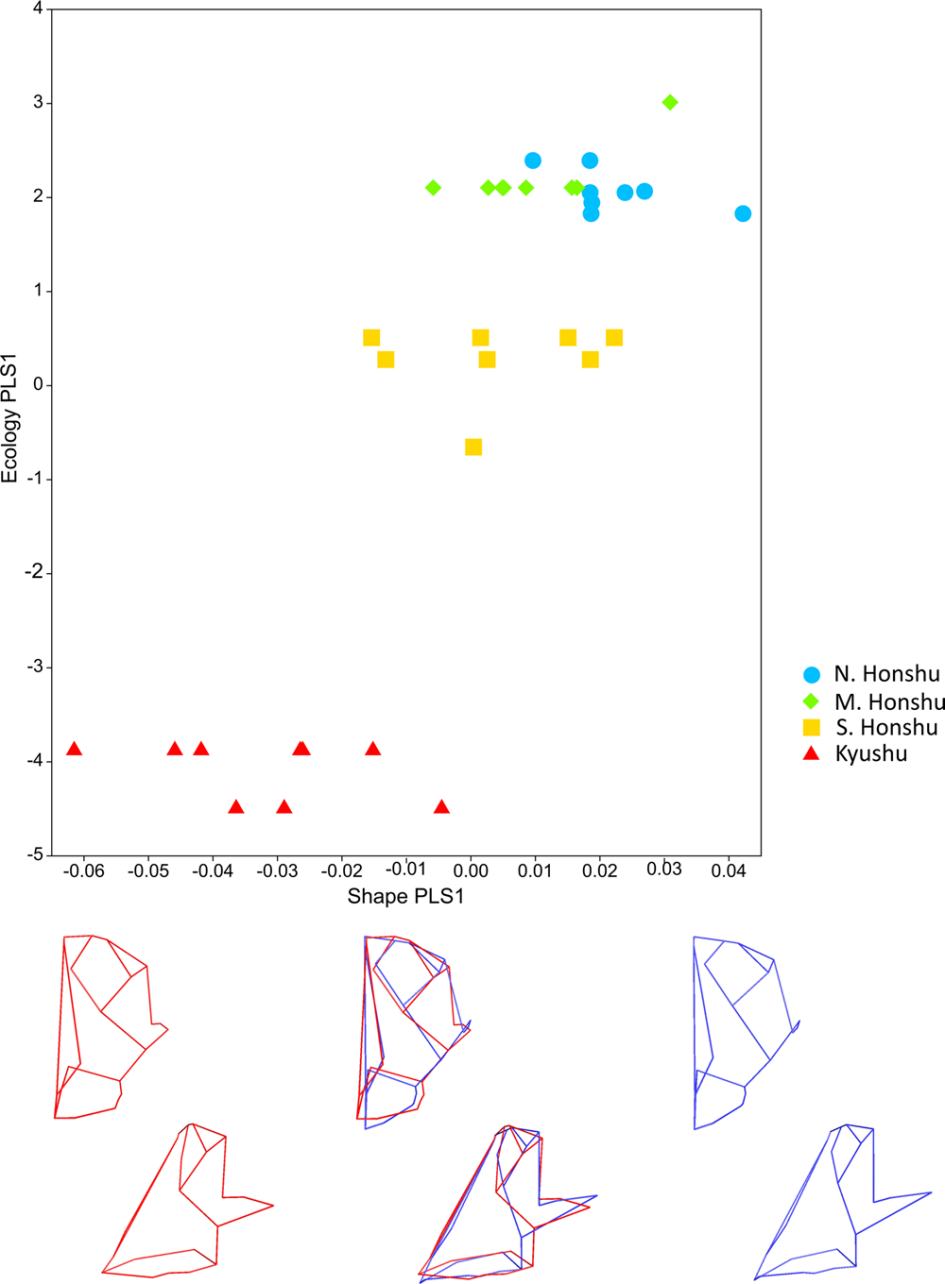
Results from 2-B PLS facial analysis. Block 1 (shape) PLS1 scores against block 2 (ecology) PLS1 scores in facial analysis of macaques (reduced sample). Points coloured by latitude group: light blue circle: N. Honshu, green diamonds: M. Honshu, yellow squares: S. Honshu, red triangles: Kyushu. Mean shape warped to show variation along shape PLS (−0.065 to 0.05 Procrustes distance) (minimum: red, maximum: blue) from front (top), and side (bottom) views. Wireframes as shown in Fig. 6.

In the reduced sample of macaques there is a moderate, significant association between blocks of facial shape and ecological data (RV coefficient: 0.27, p < 0.001). The singular value and correlation show that the relationship between blocks 1 and 2 on PLS1 (>92% covariation between blocks) are also strong and significant (singular value = 0.05, p < 0.001, correlation = 0.83, p < 0.001). As with the craniofacial analysis, there is an almost complete overlap between northern and mid-Honshu groups on PLS1, but southern Honshu and Kyushu groups are more differentiated, particularly in the case of the latter (Fig. 2).

All temperature and precipitation variables contribute fairly evenly to PLS1 (from maximum precipitation at −0.40 to temperature range at 0.35), whilst altitude loads the PLS much less strongly (Table S2.1.1). Higher scoring individuals on the ecological factor of PLS1 experience lower temperatures/levels of precipitation and greater annual temperature range (Fig. 2). On the shape factor of PLS1, these same high-scoring specimens show cheek regions that are mediolaterally broader and with more anteriorly placed zygomatics. Toothrows are narrower, and faces less projecting (Fig. 2). The similarities between the craniofacial and facial analyses suggest that for the macaques, a weaker ecological signal in the neurocranium is not masking a stronger one in the face (see below). This variation in shape is significantly different between latitude groups; as with the craniofacial landmark set, in the facial landmark set there are significant differences in block 1 PLS1 scores between all groups except mid Honshu and both north and south Honshu (Section S2.1, Table S2.1.2).

#### Jomon

In the Jomon there is no significant association between blocks of ecology and facial shape data (RV coefficient: 0.17, p > 0.05), nor do any of the individual pairs of PLS dimensions reach significance (p values > 0.05) (Tables S2.2.3 and 2.2.4 and Fig. S2.2.2).

### Covariation between neurocranial shape and ecology

#### Macaques

**Figure 3:**
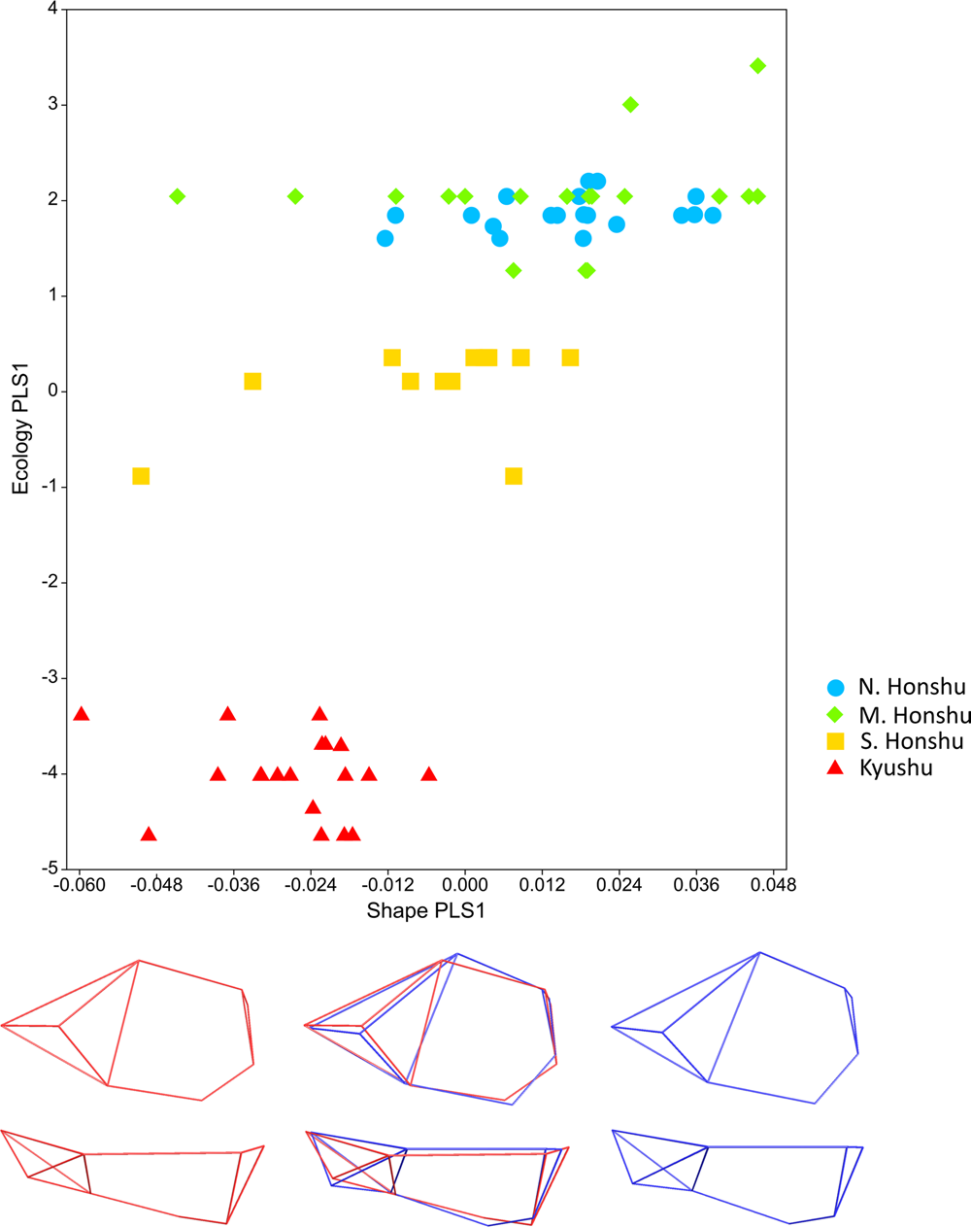
Results from 2-B PLS neurocranial analysis. Block 1 (shape) PLS1 scores against block 2 (ecology) PLS1 scores in neurocranial analysis of macaques (full sample). Points coloured by latitude group: light blue circles: N. Honshu, green diamonds: M. Honshu, yellow squares: S. Honshu, red triangles: Kyushu. Mean shape warped to show variation along shape PLS (−0.065 to 0.048 Procrustes distance) (minimum: red, maximum: blue) from side (top), and top (bottom) views. Wireframes as shown in Fig. 6.

In this analysis the full macaque sample (n = 72) was used (Table S1.1.1) to compare with the full Jomon sample (n = 83). There is a significant association between blocks of ecology and shape data (RV coefficient:  0.27, p < 0.0001). The singular value and correlation for blocks 1 and 2 on PLS1 (>93% variation between blocks) are also strong and significant (singular value = 0.05, p < 0.0001, correlation = 0.732, p < 0.0001). As for the craniofacial and facial analyses, all temperature and precipitation variables contribute fairly evenly to the block 2 component of PLS1 (mean temperature at −0.39 to temperature range at 0.36), altitude much less so (Table S2.1.1). Higher scoring specimens (Fig. 3) share lower levels of precipitation/temperatures and greater annual temperature ranges.

The shape component of PLS1 shows a shift from a more dolichocephalic to a more brachycephalic neurocranium, going from low to high scores. Neurocrania of higher scoring individuals are mediolaterally broader and superinferiorly taller (Fig. 3). This variation in shape is significantly different between latitude groups; there are significant differences in block 1 PLS1 scores between all groups except mid Honshu and north Honshu (Section S2.1, Table S2.1.2).

#### Jomon

There is no significant association between blocks of ecology and neurocranial shape data in the Jomon (RV coefficient: 0.03, p > 0.05), nor do any of the individual pairs of PLS dimensions reach significance (p values > 0.05) (Table S2.2.5 and 2.2.6, Fig. S2.2.3).

## Discussion

There is no greater variation, as measured by deviation from the mean, in cranial shape within the macaques than within the Jomon, yet there is a far stronger relationship between that variation and ecogeography in the macaques. Here we have investigated raw intraspecific variation and found it to be comparable between humans and macaques. Given considerable sexual dimorphism in macaques, a high proportion of intraspecific variation in this species is likely to be due to the differences between males and females. This suggests that there are two major sources of intraspecific variation: sex and ecology, which have strong effects on macaque morphology and little effect on human morphology. Thus, despite the similar mean Procrustes distances from centroid shape for both species, it seems likely that more of this variation is neutral in the human crania in the sample.

For all three cranial regions in macaques there is consistent, substantial covariation between shape and ecological data. In each analysis, all the temperature and precipitation variables contribute similarly and fairly equally to the relationship with shape, whilst for all of them altitude plays a secondary role. The craniofacial and facial analyses together show that macaques from colder, drier, more variable environments have broader, more anteriorly projecting cheek regions, narrower toothrows, narrower nasal apertures and less projecting faces. The craniofacial and neurocranial analyses together show these same macaques also have relatively more globular neurocrania.

In our previous research^12^, we also found that broader, flatter faces, narrower nasal apertures and rounder neurocrania were associated with more northern Japanese macaques. In that study we used the same sample but a much larger landmark set and thus higher quality shape data, and canonical variates analysis on latitude groups, rather than PLS analysis on ecological variables. The macaque morphology we see associated with colder, drier habitats in both studies mirrors the morphology seen in extremely high latitude human populations^9,16–18^, as well as in other cold-adapted non-primate mammals^20,25^. This morphology is argued to be functionally related to minimising heat loss and accommodating nasal apparatus adapted to air conditioning in cold, dry climates. The similarity between the morphology of macaques from cold, dry environments and cold-adapted humans and other mammals suggests that the macaque morphology can also be interpreted as cold-adaptation via the same functional mechanisms. The relative importance of compliance with Allen’s rule, versus responding to adaptation in the nasal apparatus, in shaping ecogeographic differences in macaque morphology could be fruitfully investigated by extending the analyses presented here to include the internal nasal morphology. The repetition of the same pattern of cold-associated morphology in all three analyses in this study, and in our previous work^12^, reinforces the strength of the pattern of association between this specific macaque morphology and a colder, more arid climate with greater annual temperature range.

In contrast to the macaque results, none of the Jomon analyses showed any significant covariation between ecological variables and shape. This is despite our use of several analyses in concert to try to maximise the chance of finding an ecogeographic pattern. In addition to our analyses of global craniofacial shape, we analysed differences in facial shape as this region is thought to show the strongest climatic signal in humans e.g., ^41^, and we analysed neurocranial shape to enlarge the sample as far as possible given the fragmentary Jomon remains. Our results, based on a systematic shape analysis of Jomon from throughout Japan, contribute to the on-going discussion of the covariates of Jomon cranial variation^30–33^. In contrast to some previous research^30–32^, we found that compared to geographically sympatric non-human primates the Jomon in this sample are not particularly morphologically homogenous (although it remains possible they are relatively homogenous compared to other human groups); however, the variation present is not patterned ecogeographically. Whilst the majority of human cranial variation outside of latitudinal extremes appears to be due to neutral processes^9^, some Jomon variation at the regional level may also be due to functional differences in diet^42^.

The Jomon era was long-lived (~13,000 years) and is subdivided into chronological periods based on ceramic style^29^. There are known differences in material culture, subsistence, demography and morphology between these periods^28,43–46^. Temple^45^ found that Jomon stature decreases over time in western Japan, probably due to stunting resulting from changes in climate and population density and associated dietary shifts^45^. Hoover and Williams also noted differences in some mandibular measurements between time periods^42^. Out of necessity due to our archaeological sample, we included remains from Jomon sites based on availability, an aim to balance the sexes and cranial completeness, as well as site location and environment. We were not able to collect a data set of reasonable geographic breath and size from any single time period and the sites we included date to between ~6000-2000 BP, with many sites also having wide potential date ranges (Table S2.3.1). This spread in time means that chronological differences in morphology may be present and could conceivably obscure ecogeographic patterns. The cranial morphology we analyse here is less likely to be affected by dietary stress than the limb lengths reported by Temple^45^, however, as skeletal regions are differentially plastic to environmental change, with the diaphyses of the long bones being amongst the most plastic and the cranium the least^36,37^. This low likelihood is further supported by Hoover and Williams’ research, which shows that although there are differences in mandibular shape between time periods, these are insubstantial compared to differences between regions^42^.

During the Holocene era, climate has been more stable than the long time-scale glacial/interglacial cycles and shorter-term, but sometimes extreme, fluctuations of the preceding Pleistocene^47^. Nonetheless, there has still been global and regional climate change during the Holocene^48^. After the cold of the Younger Dryas climate deterioration (~13,000 BP), during which Japan was 6–9 °C colder than present depending on region, the archipelago experienced warming through the early part of the Holocene^49^. The peak of this trend was mid Holocene (8,200-3,300 BP) with a period of temperatures 1–2 °C warmer than present Japanese temperatures, followed by a gradual cooling from ~3000 BP to present^49^. The Mid-Holocene climatic optimum overlaps with the majority of Jomon culture, from the Initial to the Late Period^29^ and all of the Jomon sites in the current sample have chronologies where at least part of the estimated date range (Table S2.3.1) would be within the Mid-Holocene period^49^. Due to the potential disparity between current climate and that experienced by the Jomon sample during their lives, we tested the covariation between cranial shape and estimated palaeoclimatic variables (WorldClim Mid-Holocene climate estimation, ~6,000 BP^50^). As with the current climate variables, the relationships between palaeoclimate and cranial shape in all analyses were non-significant (SI section S2.3), this suggests that the lack of an ecogeographic pattern in Jomon cranial morphology is robust and not an artefact of a mismatch between palaeo- and current climates.

If the ecological threshold required for adaptation and rates of adaptation are similar across primates, an arguable explanation for a lack of climatic signal in the Jomon could be an earlier arrival in Japan by macaques than humans. The oldest macaque fossil in Japan is dated to 630,000-430,000 BP^51^, but molecular evidence suggests that the present radiation is much more recent, with northern populations having gone extinct during the last glaciation and these regions only having been recolonised from coastal and low-land populations from mid Honshu <15,000 BP^52,53^. It is possible there was some clinal variation within the macaques which survived the last glaciation in mid-to-South Japan, potentially giving the northernmost survivors a ‘head-start’ on adapting to higher latitudes following climatic amelioration, although this is not visible from the molecular data^54^. Although the earliest Jomon sites date to ~15,000 BP^29^, the oldest human remains in Japan are dated to ~30,000 BP^55^. There is debate over the extent of continuity between the Neolithic Jomon and the preceding Palaeolithic population(s) of Japan^55^, but there are apparent morphological similarities between the Jomon and some of the earliest well-preserved Japanese inhabitants, dated to ~30-15,000 BP^56–58^. The Jomon therefore, can be said to have at least some input from populations with a history in Japan dating back to ~30,000 BP. Irrespective of potential differences in the deep timespans of Japanese occupation between the species, studies of translocation of macaques between habitats^59^ and of humans emigrating to new countries^60^ show that adaptation to local conditions can occur within the course of a few generations. On this time-scale it can be argued that both species have had more than sufficient time to adapt to their environments.

Extant Japanese macaques live in islands of suitable habitat surrounded by urban development and agricultural land^61^. This habitat fragmentation makes geneflow between groups as widely separated as those in this study very unlikely. The archaeological evidence for the Jomon on the other hand, suggests both deep regional differences, as shown by patterns of ceramic style^44,62^ and tooth evulsion^63^, and sufficient contact between groups to maintain a cohesive culture with evidence of long-distance trade networks throughout the archipelago^62^. Commonality of culture and movement of people are likely to have been accompanied by geneflow in the Jomon, which could have contributed to the lack of a relationship between cranial morphology and climate found here. It should be noted however, that the Jomon are not more similar to one another than the macaques, showing very similar amounts of intraspecific variation. Additionally, ancient DNA analyses of Jomon remains to date show a complicated picture, supporting only limited geneflow between groups separated by long distances^64,65^. This argues against a strong effect of geneflow on the patterns of Jomon cranial morphology throughout this sample, but future analyses of morphology within a framework of explicit hypotheses of neutrality could better clarify the roles of drift and geneflow within the Jomon.

An obvious difference in the way in which humans adapt to their environments in comparison with other primates is the degree to which we depend on behavioural strategies. Humans may be buffered by their cultural and behavioural adaptations, which mediate the relationship between environment and skeletal morphology^38,39,66^. Jomon culture was complex, including sophisticated housing, clothing, hunting/food processing/food storage technology, fire, ritual beliefs and items^29,62^. Local behaviour and material culture were well-adapted to the various environmental requirements of the different constituent ecosystems of the Japanese archipelago^28,29^. These innovations likely mediated local stressors and in turn even shaped regional environments to suit their human inhabitants, freeing them from the need to adapt physically (at least in terms of skeletal morphology). This constitutes a mechanism by which the Jomon adapted to their environment that is unavailable to the geographically sympatric macaque groups and this difference between humans and other primates potentially reflects a strategy of adaptive flexibility that was important in the wider success of human colonisation of novel habitats during human evolution and expansion^1–3^.

## Conclusions

There is comparable intraspecific variation in cranial morphology between samples of macaques and of Jomon throughout Japan. In macaques, however, this variation covaries strongly and consistently with climate, whereas this is not the case for the Jomon. Macaque morphology associated with colder, drier, more variable climates fits expectations of mammalian cranial cold-adaptation, suggesting that ecogeographic patterns in macaque cranial shape show intraspecific functional adaptation to climate. There is no covariation between ecology and cranial morphology in the Jomon sampled in this study. Although *H*. *sapiens* as a species seems to follow the general primate pattern of climatic adaptation^12^, in the regional example of the Jomon, humans seem to exhibit less cranial adaptation to comparable climatic stresses. If the apparent differences in cranial climatic adaptation between geographically sympatric humans and non-humans primates are borne out by future investigations, they may be linked to greater reliance on behavioural adaptation in humans^2,3,38,39^. Our findings re-emphasise the importance of behavioural strategies in human evolution and dispersal and may shed light on the reasons for the survival of *H*. *sapiens* to the exclusion of all other hominin species^2,3^.

## Materials and Methods

### Materials

#### Sample

**Figure 4:**
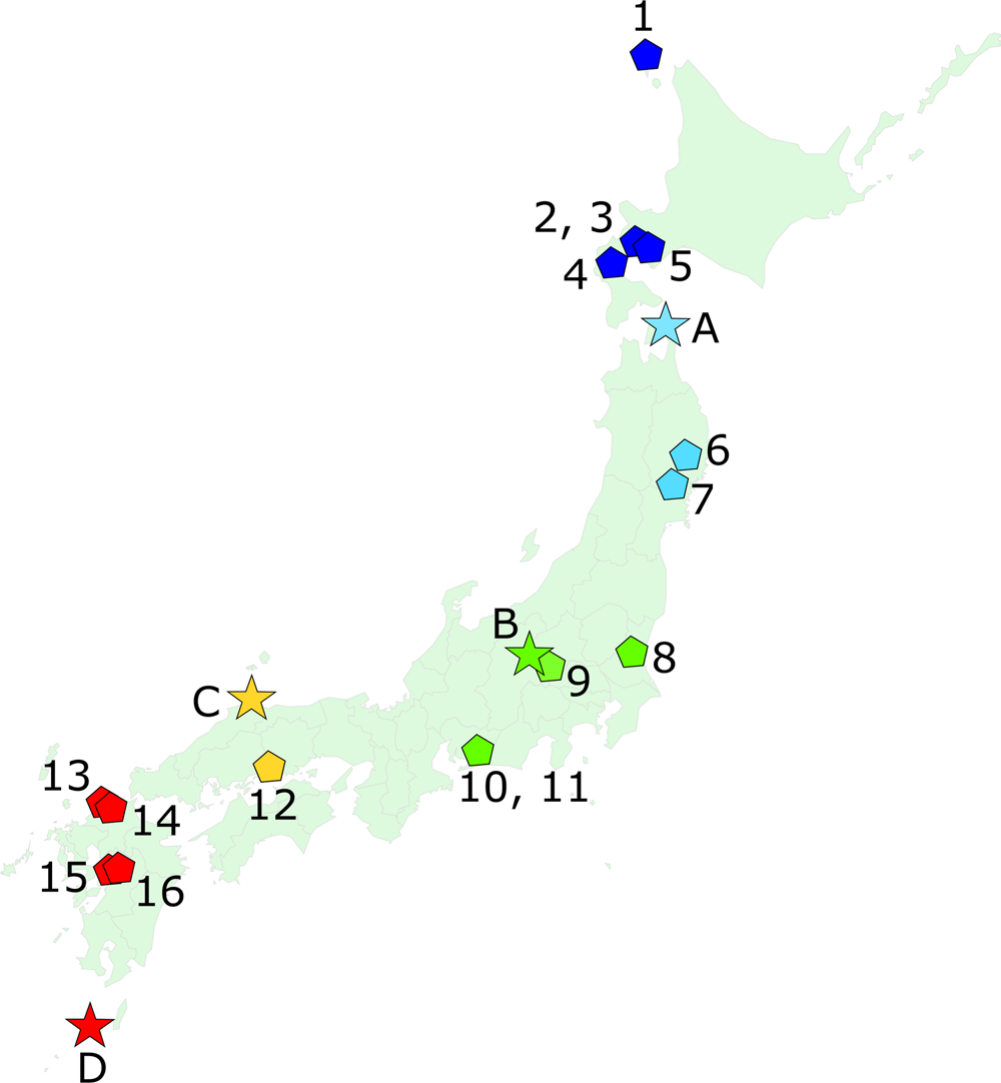
Map showing origins of macaque (stars/letters) and Jomon (pentagons/numbers) samples. Colour coding refers to geographic grouping used in paper. Top right to bottom left, dark blue: Hokkaido, pale blue: N. Honshu, green: M. Honshu, yellow: S. Honshu, red: Kyushu. A: Shimokita, B: Nagano, C: Shimane, D: Yakushima. 1: Funadomari, 2: Takasago, 3: Irie, 4: Kotan Onsen, 5: Kitakogane, 6: Miyano, 7: Ebishima, 8: Wakaumi, 9: Tochibara, 10: Yoshigo, 11: Ikawazu, 12: Tuskumo, 13: Yamaga, 14: Einomaru, 15: Todoroki, 16: Goryo.

The macaque sample (Table S1.1.1, Fig. 4) consisted of 72 wild-shot, adult Japanese macaque crania from the collection housed at the Primate Research Institute, Kyoto University (PRI). This is a subsample of that used in our recent paper on Japanese macaque ecogeography^12^, where more details can be found. The specimens come from locations at four different latitudes within Japan: Shimokita in North Honshu; Nagano in mid Honshu; Shimane in South Honshu and Yakushima Island off the southern coast of Kyushu (Fig. 4). This sample includes both the most northerly and most southerly extant populations of *M*. *fuscata* in the wild. We CT scanned the macaque crania using the Asteion Premium 4 helical scanner (Toshiba Medical Systems, Otawara, Japan) housed at the PRI. Voxel sizes vary slightly between individuals due to differences in cranial size, but all are approximately 0.3 mm × 0.3 mm with a slice thickness of 0.5 mm. We produced virtual mesh surfaces (.ply) from CT data in AVIZO 9.1 (FEI, Hilsboro, USA) for digitising.

The Jomon sample comes from archaeological sites of Jomon age and culture throughout Japan (Fig. 4, Tables S1.1.2, S1.1.3 and S2.3.1) and consists of material housed at the institutions listed in the Acknowledgements. Where possible (Kyoto University only) we CT scanned crania using an Alexion TSX-032A (Toshiba Medical Systems, Otawara, Japan) scanner at their housing institution. We optimised scan parameters for individual specimens, which varied slightly in size, but all voxel sizes are ~0.4 mm × 0.4 mm with a slice thickness of 1 mm. We created surface models from these data in AVIZO as for the macaques. Where no CT scanner was available we surface scanned the crania using an Artec Space Spider structured light scanner (Artec 3D, Luxenbourg) with a maximum resolution of 0.1 mm. One individual from Einomaru (Kyushu) was scanned using a Next Engine laser scanner (Next Engine, Santa Monica, USA) with a maximum resolution of 0.3 mm. Whilst combining data generated different methods may be an additional source of error, in this case it was necessary due to time and equipment constraints and the need to preserve sample size. Recent studies have shown that error resulting from the use of different types of scanning technology is generally minor compared to biological signals in the data^67,68^. We cleaned, aligned and fused scans in Artec/Next Engine proprietary software and exported surface models to AVIZO as.ply files for digitising.

#### Ecological variables

We obtained ecological variables (Table S1.2.4) from the WorldClim database^50^, matching climate and altitude records to site locations (longitude and latitude), and extracted the data using ArcGIS release 10^69^ and DIVA GIS version 1.4^50^. For macaques with unknown exact locations (located only to prefecture in the PRI records), we used means of ecological variables from other sites with known exact locations in that group. The ecological variables we used were mean annual temperature (MeanTemp), maximum temperature of the warmest month (MaxTemp), minimum temperature of the coldest month (MinTemp), annual temperature range (MaxTemp – MinTemp: TempRange), annual precipitation (AnnPrecip), precipitation during the wettest month (MaxPrecip), precipitation during the driest month (MinPrecip) and Altitude. When referring to all these variables together we use the term ecological, since altitude is not a measure of climate albeit closely related; when referring to the temperature and precipitation variables to the exclusion of altitude, we use the term climatic. We transformed ecological variables into Z scores to enable the inclusion of variables measured in different units in the same partial least squares analysis. In some analyses to ease visualisation and comparison with our earlier work on Japanese macaque ecogeography^12^, we also divided the sample into geographic groups as follows: Hokkaido, North Honshu, mid Honshu, South Honshu, and Kyushu (Fig. 4). There is no Hokkaido group for the macaques as they have never inhabited this island.

### Methods

#### Landmarks

The surface models of crania were digitised by a single observer in AVIZO 9.1. We used unilateral landmarks to remove the noise of bilateral asymmetry and to allow mirroring of bilateral landmarks preserved on only one side, thus increasing sample size. We mirrored missing bilateral landmarks in Morpheus^70^ using a midline calculated using all landmarks present. We only reflected missing landmarks in cases where fewer than four landmarks were missing, to ensure sufficient information on the midline for accurate reflection. We used either right or left sides of crania, depending on which side was most complete, these were then reflected to the side of the first specimen in the sample during Procrustes generalised superimposition in MorphoJ^71^.

The landmarks are subsets of those used in Buck *et al*.^12^ to show ecogeographic patterns in macaque cranial morphology. We used only landmarks that could be placed on both macaque and human crania and we chose them with the aim of balancing the opposing needs of capturing detailed cranial shape and optimising sample size in the archaeological, and often fragmentary, Jomon sample. To this end, we used three landmark sets with different sample sizes: craniofacial, facial and neurocranial landmark sets. Note that, throughout the paper we use the term cranial to refer to shape across all regions, i.e., craniofacial, facial and neurocranial, whilst we refer to the individual regional analyses by name.

**Figure 5:**
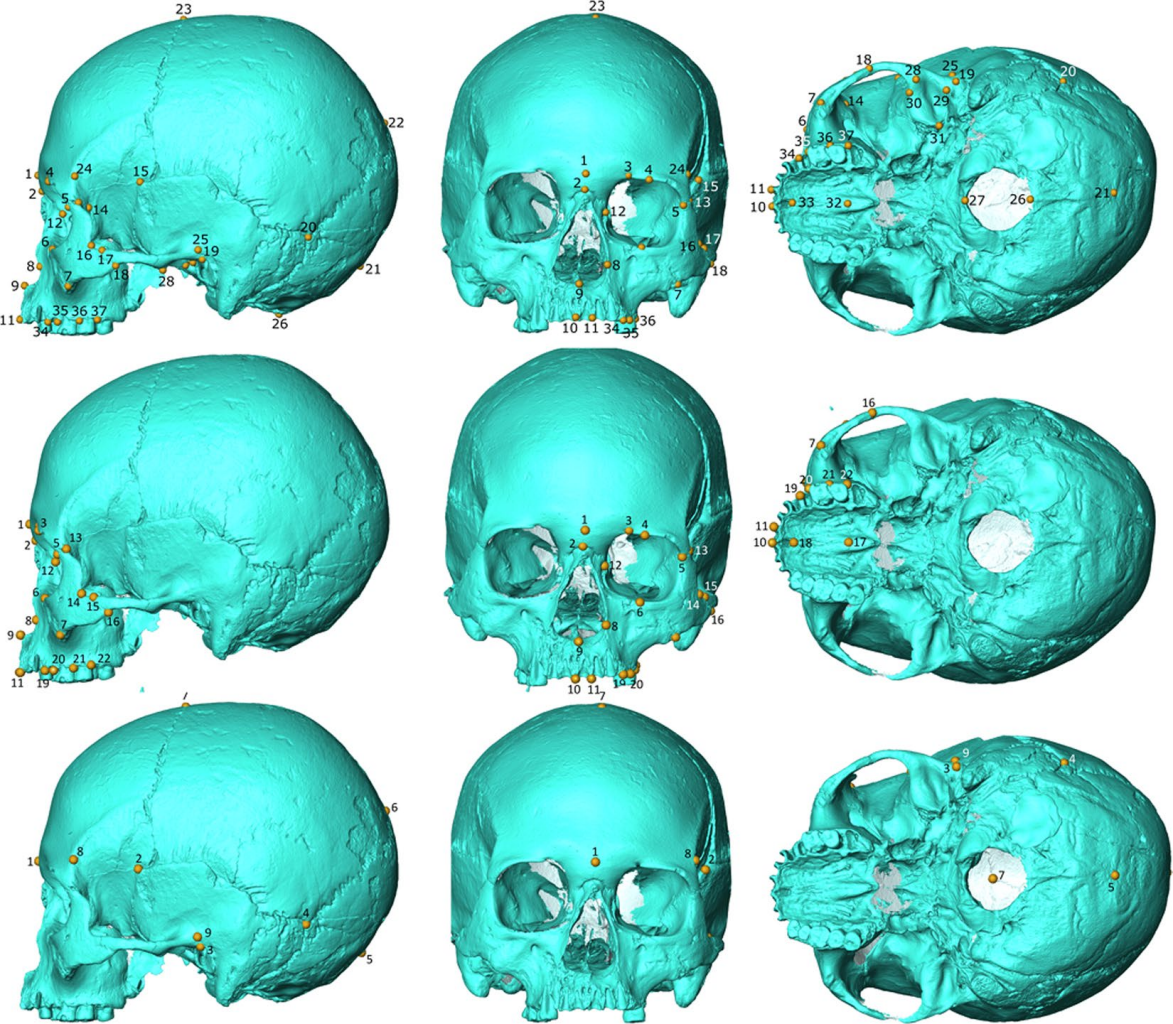
Landmarks for craniofacial (top), facial (middle) and neurocranial (bottom) landmark sets seen from side (left), front (middle) and bottom (right). Jomon from Takasago (Hokkaido) used for illustration.

The craniofacial landmark set (Table S1.2.1, Fig. 5) consisted of 37 landmarks distributed across the ectocranial surface of the cranium, but allowed the inclusion of only 33 Jomon (Table S1.1.2), thus optimising data quality at the expense of sample size. The facial landmark set (Table S1.2.2, Fig. 5) was a subset of the craniofacial landmark set, using landmarks located on the face, and it included the same individuals. Despite the lower landmark number, it was not possible to include any more specimens as the face is one of the most delicate regions of the cranium and therefore one of the most frequently damaged. We chose to investigate a facial landmark set in addition to the craniofacial landmark set as it has been previously reported that the face is the cranial region showing the strongest climatic signal^41^. The neurocranial landmark set (Table S1.2.3, Fig. 5) consisted of nine landmarks describing the shape of the neurocranium and allowed us to include 83 Jomon specimens (Table S1.1.3), thus maximising sample size over shape detail. Despite the potentially stronger climatic signal in the face, previous research indicates there are also climatic differences in neurocranial shape between groups of *H*. *sapiens*, particularly when very high latitude groups are included e.g., ^14^. As the macaques are recent and wild-shot they are very complete, we were therefore able to conduct all analyses on the full sample of 72 individuals where appropriate. We also used a reduced, randomly chosen, sample of 33 macaques (equal numbers from each site), for direct comparisons between macaque and Jomon craniofacial/facial analyses (n = 33) (Table S1.1.1).

**Figure 6:**
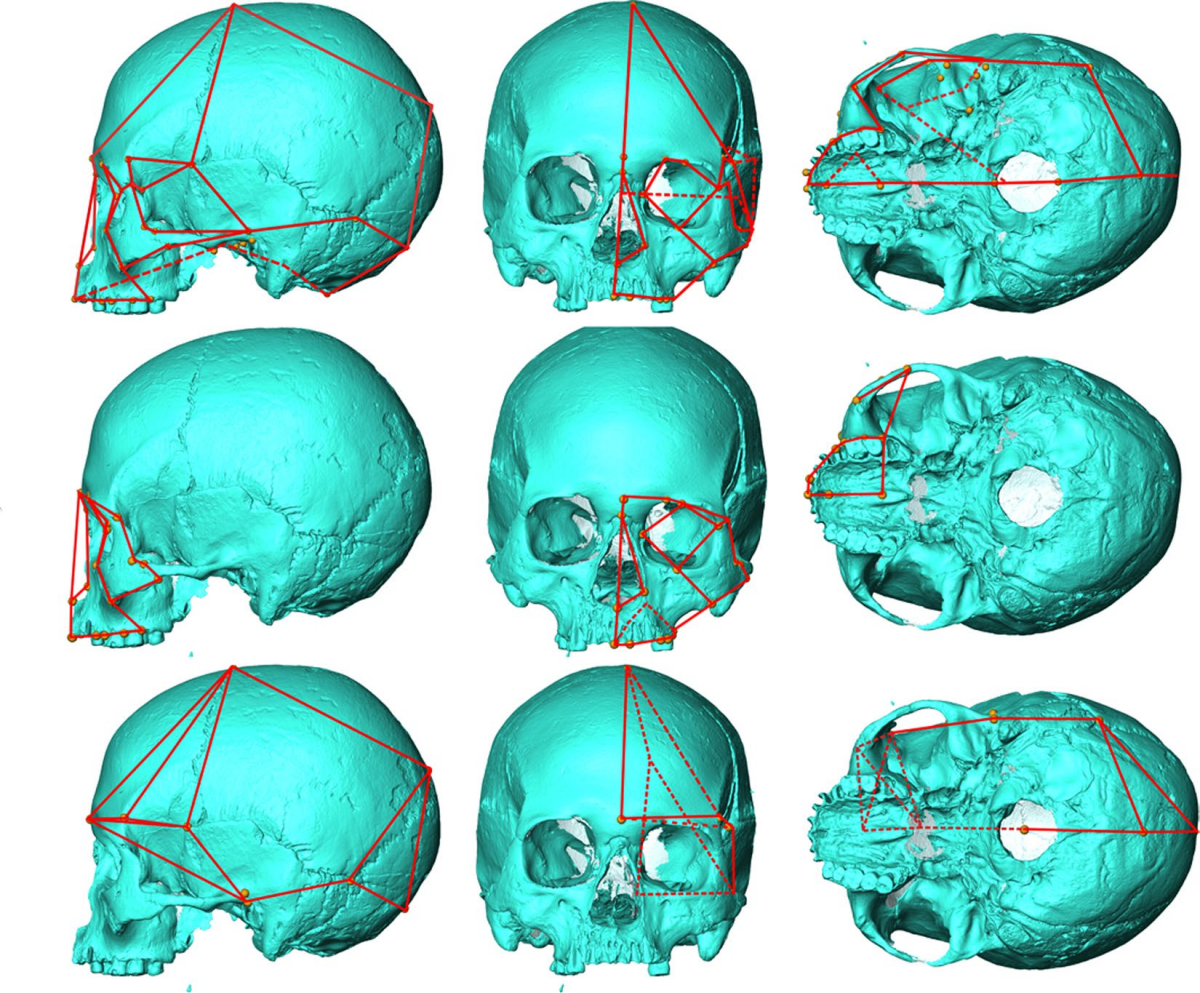
Wireframes used in visualising shape changes in craniofacial (top), facial (middle) and neurocranial (bottom) analyses from side (left), front (middle) and bottom (right) views. Dotted lines show connections between landmarks hidden by cranium. Some lines not shown in views from below for clarity. Jomon from Takasago (Hokkaido) used for illustration.

#### Analyses

We exported coordinates from landmarks digitised in AVIZO to MorphoJ^71^ and subjected them to generalised Procrustes superimposition to remove the effects of translation, rotation and (non-allometric) size differences. To use the suite of statistical tools designed for Euclidean space, we projected the landmark configurations from shape space into a tangent space. To remove the effect of the large degree of sexual dimorphism in Japanese macaques, the partial least squares analyses (see below) were carried out on the residuals from multiple regression analyses of sex on shape for each landmark set. Despite the predictions of Bergmann’s rule, the relationship between size and ecology was not investigated in addition to that between shape and ecology because previous research has shown that probable insular dwarfism in Yakushima macaques masks any potential ecogeographic signal in size in this sample^12^

We conducted two-block partial least squares analyses (2B-PLS) in MorphoJ to investigate the covariation between blocks consisting of different types of multivariate, continuous data. Block one contained shape data (Procrustes coordinates) and block two ecological data (temperature, precipitation and altitude). 2B-PLS finds new pairs of variables (PLS dimensions), one from each block, that maximise the covariance between blocks. Each PLS dimension explains successively less of the covariation between blocks in a manner analogous to principle components analysis. Explanatory factors on each PLS are correlated only with each other and are orthogonal to all other PLS dimensions. The two blocks for a particular PLS dimension can be plotted against one another to interpret the change in shape score per change in ecological score. By warping mean shapes it is possible to model the shape differences represented by the Block 1 factor of each PLS to observe their relationship with the Block 2 factor (ecology). The analysis provides an RV coefficient, a measure of the strength of the overall association between the two blocks, and singular values and correlation coefficients for each PLS, which are measures of the strength of the relationship between the two blocks for that PLS. It also produces loadings that show the importance of each variable to the covariation between the two blocks. Comparison between different PLS analyses is inadvisable because the RV coefficient is affected by sample size and the number of variables such that smaller samples and higher numbers of variables both inflate the RV coefficient^72^. For that reason, here direct comparison between PLS analyses is made only when the sample sizes are the same, or very similar, and when the variables (i.e., the number of landmarks and of ecological variables) are the same. This is the reason for the reduced sample size in macaques, which allows direct comparison with the craniofacial/facial sample of Jomon (Table S1.1.1).

To investigate the amount of shape variation within each sample we examined the Procrustes distances between individuals in shape space^73,74^. Using Morphologika^75^, we calculated the Procrustes distances between individuals and their species centroids and then calculated their means, standard deviations and ranges. The mean and standard deviations of Procrustes distances are informative regarding the dispersal of the data around their centroid, greater morphological variation within a group leading to greater dispersal around the mean. The range in Procrustes distances between individuals and the species centroid indicates whether certain individuals in the sample are anomalous and affecting the mean. If climates are comparable between species, a greater amount of adaptation to a stressor in a sample could be expected to lead to a greater amount of variation between conspecific individuals differentially experiencing that stressor. For example, greater adaptation to a particular level of cold-stress would lead to a greater morphological difference between individuals from warm climates and cold climates, and thus larger mean Procrustes distances.

## Supplementary information

Supplemental information
