# Supplementary material for: Evidence of different climatic adaptation strategies in humans and non-human primates
Source: Sci Rep. 2019 Jul 30;9:11025. doi: 10.1038/s41598-019-47202-8 (PMC6667491; doi:10.1038/s41598-019-47202-8)
Supplement: Supplementary file 1 — Supplemental information [file 41598_2019_47202_MOESM1_ESM.pdf]

Evidence of different climatic adaptation strategies in humans and non-human primates

Buck, L. T.[1, 2,3 *], De Groote, I.[4], Hamada, Y.[5], Hassett, B. R.[6,2], Ito, T.[5] and Stock, J. T.[1, 7, 8]

[1.] PAVE research group, Department of Archaeology, University of Cambridge, Pembroke Street, Cambridge, CB2 3QG, UK.
[2.] Human Origins Research Group, Department of Earth Sciences, Natural History Museum, Cromwell Road, London, SW7 5BD, UK.
[3.] Department of Anthropology, University of California Davis, 1 Shields Avenue, Davis, CA, 95616, USA.
[4.] School of Natural Science and Psychology, Liverpool John Moores University, James Parsons Building, Byrom Street, Liverpool, L3 3AF, UK.
[5.] Primate Research Institute, Kyoto University, Inuyama, Aichi, 484-8506, Japan.
[6.] Institute of Archaeology, University College London, 31-4 Gordon Square, London, WC1H 0PY, UK.
[7.]Department of Anthropology, Western University, London, Ontario, Canada, N6A 3K7
[8.] Department of Archaeology, Max Planck Institute for the Science of Human History, Kahlaische Strasse 10, D-07745 Jena, Germany.

* Corresponding author. Email: lbuck@ucdavis.edu.

Contents

S1. Materials and Methods
S1.1 Materials

S1.2 Methods

S2. Results
S2.1 Macaque analyses

S2.2 Jomon analyses: current ecological variables

S2.3 Jomon analyses: palaeoclimatic variables

S.3 References

S1. Materials and methods

S1.1. Materials

Table S1.1.1: Macaque full sample of 72 crania and reduced sample of 33. F: female, M: male.

Group	Prefecture	Sex	Full sample n	Reduced sample n
N. Honshu	Shimokita	F	10	4
		M	9	4
M. Honshu	Nagano	F	9	4
		M	10	4
S. Honshu	Shimane	F	10	4
		M	4	4
Kyushu	Yakushima	F	10	4
		M	10	5

Table S1.1.2: Jomon craniofacial and facial sample of 33. F: female, M: male, U: sex undiagnosed.

Group	Site	Sex	Latitude	n	Group total
Hokkaido	Funadomari	F	45.35	1	
		M		1	
		U		0	
	Kitakogane	F	42.54	0	
		M		2	
		U		0	
	Kotan Onsen	F	43.57	1	
		M		1	
		U		0	
	Takasago	F	42.55	1	
		M		1	
		U		1	9
N. Honshu	Ebishima	F	39.94	1	
		M		4	
		U		0	
	Miyano	F	39.05	0	
		M		2	
		U		0	7
M. Honshu	Yoshigo	F	34.67	1	
		M		1	
		U		0	
	Wakaumi	F	36.12	0	
		M		1	
		U		0	
	Tochibara	F	36.06	1	
		M		0	
		U		0	
	Ikawazu	F	34.67	0	
		M		1	
		U		0	5
S. Honshu	Tuskumo	F	34.50	5	

		M		4	
		U		0	9
Kyushu	Goryo	F	32.50	0	
		M		1	
		U		0	
	Yamaga	F	33.58	2	
		M		0	
		U		0	3

Table S1.1.3: Jomon neurocranial sample of 83. F: female, M: male, U: sex undiagnosed.

Group	Site	Sex	n	Group total
Hokkaido	Funadomari	F	2	
		M	2	
		U	1	
	Irie	F	0	
		M	1	
		U	1	
	Kitakogane	F	1	
		M	2	
		U	1	
	Kotan Onsen	F	1	
		M	2	
	Takasago	F	1	
		M	2	
		U	1	18
N. Honshu	Ebishima	F	9	
		M	8	
	Miyano	F	0	
		M	3	20
M. Honshu	Yoshigo	F	6	
		M	5	
	Wakaumi	F	0	
		M	1	
	Tochibara	F	3	
		M	0	
	Ikawazu	F	1	
		M	2	
		U	1	19
S. Honshu	Tuskumo	F	10	
		M	9	
		U	1	20
Kyushu	Einomaru	F	1	
		M	0	
	Goryo	F	0	
		M	1	
	Todoroki	F	1	
		M	0	

	Yamaga	F	2	
		M	1	6

S.1.2 Methods

Table S1.2.1: Craniofacial landmark set of 37 landmarks.

Name	Definition	Number in craniofacial landmark set
Glabella	Most anterior midline point on frontal	1
Nasion	Meeting point of nasals and frontal	2
Supraorbital notch	Most lateral point on supraorbital notch/foramen	3
Mid-torus inf.	Point on inferior margin of supraorbital torus (superior margin of orbit) roughly at middle of orbit	4
Frontomalare orbital	Meeting point of frontozygomatic suture and orbital margin	5
Zygoorbitale	Meeting point of zygomaxillary suture and orbital margin	6
Zygomaxillare	Most inferior point on zygomaxillary suture	7
Alare	Most lateral point on nasal margin	8
Nasiospinale	Most anteroinferior point of piriform aperture	9
Prosthion	Most inferior point on alveolar bone between central incisors	10
Prosthion 2	Most inferior point on alveolar bone between central and lateral incisors	11
Dacryon	meeting point of frontal, maxilla and lacrimal	12
Frontomalare temporale	Most lateral point on frontozygomatic suture	13
Zygomatic arch / alisphenoid / frontal	Meeting point of zygomatic arch, alisphenoid and frontal bone	14
Pterion pos.	Meeting point of frontal, parietal and sphenoid	15
Zygomatic arch ant.	Maximum curvature of anterior upper margin of zygomatic arch	16
Zygotemporale sup.	Most superior point on zygotemporal suture	17
Zygotemporale inf.	Most inferior point on zygotemporal suture	18
Porion	Most superior point on external auditory meatus	19
Asterion	Meeting point of lambdoid, parietomastoid, and occipitomastoid sutures	20
Inion	Meeting point of superior nuchal lines	21
Lambda	Meeting point of lambdoidal and sagittal sutures	22
Bregma	Meeting point of sagittal and frontal sutures	23

Frontotemporale	Most medial point on temporal line on frontal	24
Zygomatic process pos.	Posteriormost point of zygomatic process of temporal bone	25
Opisthion	Midline point on posterior margin of foramen magnum	26
Basion	Midline point on anterior margin of foramen magnum	27
Articular tubercule	Most inferior post on articular tubercule	28
Post-glenoid process dist.	Distal most point on post-glenoid process	29
Temporal zygomatic curve pos.	Posteriormost point on curvature of anterior margin of zygomatic process of temporal	30
Petrous / alisphenoid / zygomatic	Meeting point petrous temporal, alisphenoid & base of zygomatic process of temporal	31
Maxilla / palate	Meeting point of maxilla and palatine along midline	32
Incisivion	Most posterior midline point of incisive foramen	33
P3 / 4	Contact point between P3/4 projected onto alveolar margin	34
P4 / M1	Contact point between P4/M1 projected onto alveolar margin	35
M1 / 2	Contact point between M1/2 projected onto alveolar margin	36
M2 / 3	Contact point between M2/3 projected onto alveolar margin	37

Table S1.2.2: Facial landmark set, 22 landmarks.

Name	Number in facial landmark set
Glabella	1
Nasion	2
Supraorbital notch	3
Mid-torus inf.	4
Frontomalare orbital	5
Zygoorbitale	6
Zygomaxillare	7
Alare	8
Nasiospinale	9
Prosthion	10
Prosthion 2	11
Dacryon	12
Frontomalare temporale	13
Zygomatic arch ant.	14
Zygotemporale sup.	15
Zygotemporale inf.	16
Maxilla / palate	17

Incisivion	18
P3 / 4	19
P4 / M1	20
M1 / 2	21
M2 / 3	22

Table S1.2.3: Neurocranial landmark set, 9 landmarks.

Name	Number in neurocranial landmark set
Glabella	1
Pterion pos.	2
Porion	3
Asterion	4
Inion	5
Lambda	6
Bregma	7
Frontotemporale	8
Zygomatic process pos.	9

Table S1.2.4: Ecological details for Jomon (*H. sapiens*) and macaques (*M. fuscata*). Variables from WorldClim (www.worldclim.org). Temperatures in °C, precipitation in mm, altitude in metres above sea-level. Mean temperature: annual mean temperature, maximum temperature: maximum temperature of warmest month, minimum temperature: minimum temperature of coldest month, temperature range: maximum – minimum, maximum precipitation: precipitation of wettest month, minimum precipitation of driest month. Macaque variables are means for all sites in latitude group.

Site	Species	Group	Mean temperature	Maximum temperature	Minimum temperature	Temperature range	Annual precipitation	Maximum precipitation	Minimum precipitation	Altitude
Funadomari	*H. sapiens*	Hokkaido	5.2	21.4	-10	31.4	1162	136	59	200
Irie	*H. sapiens*	Hokkaido	8.1	24.8	-7.1	31.9	1232	153	70	14
Kitakogane	*H. sapiens*	Hokkaido	8.3	24.4	-6.3	30.7	1204	157	65	13
Kotan-Onsen	*H. sapiens*	Hokkaido	8.2	24.7	-6.7	31.4	1208	155	68	5
Takasago	*H. sapiens*	Hokkaido	7.6	24.5	-8	32.5	1255	152	72	98
Ebishima	*H. sapiens*	N. Honshu	11.0	27.9	-4.6	32.5	1233	166	55	28
Miyano	*H. sapiens*	N. Honshu	9.5	26.3	-6.0	32.3	1413	196	55	303
Ikawazu	*H. sapiens*	M. Honshu	15.8	30.8	1.4	29.4	1786	249	58	25
Tochibara	*H. sapiens*	M. Honshu	7.4	24.7	-10.4	35.1	1523	208	39	1245
Wakaumi	*H. sapiens*	M. Honshu	14.0	29.5	-2.4	31.9	1386	185	46	27
Yoshigo	*H. sapiens*	M. Honshu	15.8	30.8	1.4	29.4	1786	249	58	25
Tsukumo	*H. sapiens*	S. Honshu	14.8	31.5	-0.4	31.9	1224	186	40	70
Einomaru	*H. sapiens*	Kyushu	16.0	31.4	2.1	29.3	1750	301	68	17
Goryo	*H. sapiens*	Kyushu	16.4	32.3	0.5	31.8	2014	369	59	46
Todoroki	*H. sapiens*	Kyushu	16.6	32.4	0.9	31.5	1974	362	59	23
Yamaga	*H. sapiens*	Kyushu	16.0	31.0	2.5	28.5	1759	317	68	20
Shimokito	*M. fuscata*	N. Honshu	9.2	25.91	-5.89	31.8	1261.63	169.11	71.74	131.95
Nagano	*M. fuscata*	M. Honshu	8.9	26.6	-8.6	35.19	1504.68	214.63	47.53	1054.37
Shimane	*M. fuscata*	S. Honshu	13.2	28.91	-1.55	30.46	1830.35	263.2	86.35	343.2
Yakushima	*M. fuscata*	Kyushu	15.9	27	4.59	22.41	3331.75	570.05	133.05	612.6

S2. Results

S2.1 Macaque analyses

Table S2.1.1: PLS1 coefficients for Block 2 (ecological variables) for macaque analyses for each cranial region.

Variable	Craniofacial	Facial	Neurocranial
Altitude	0.17	0.13	0.17
MeanTemp	-0.39	-0.39	-0.39
MaxTemp	-0.36	-0.36	-0.36
MinTemp	-0.38	-0.38	-0.38
TempRange	0.36	0.35	0.36
AnnPrecip	-0.39	-0.40	-0.36
MaxPrecip	-0.38	-0.40	-0.36
MinPrecip	-0.36	-0.35	-0.38

Results from ANOVAs on shape PLS scores

ANOVAs show significant differences in shape (block 1) PLS1 scores between macaque groups. For the craniofacial ($F(3, 29) = 40.86$, $p = < 0.001$) and facial ($F(3,29) = 26.16$, $p < 0.001$) landmark sets, Tukey's post-hoc tests show these to be differences between all groups except M. Honshu, which is not significantly different from N. Honshu or S. Honshu (Table 2.1.2). For the neurocranial landmark set ($F(3, 67) = 24.01$, $p < 0.001$) there are significant differences between all groups except M. and N. Honshu (Table S.2.1.2).

Table S2.1.2: Tukey's post-hoc tests on block 1 (shape) PLS1 scores for craniofacial and facial neurocranial landmark sets. Matrices are symmetrical, above the trace: p values, below the trace: Tukey's Q. *: significant at α = 0.05, **: significant at α = 0.005.

	Craniofacial				Facial					Neurocranial				
	N. Honshu	M. Honshu	S. Honshu	Kyushu		N. Honshu	M. Honshu	S. Honshu	Kyushu		N. Honshu	M. Honshu	S. Honshu	Kyushu
**N. Honshu**		0.5822	0.0032**	0.0002**	**N.Honshu**		0.2605	0.0460*	0.0002**	N.Honshu		0.9881	0.0077*	0.0002**
**M. Honshu**	1.8110		0.0688	0.0002**	**M.Honshu**	2.6530		0.8113	0.0002**	M.Honshu	0.4603		0.0195*	0.0002**
**S. Honshu**	5.4580**	3.6470		0.0002**	**S.Honshu**	3.9080*	1.2550		0.0002**	S.Honshu	4.7030*	4.2430*		0.0017**
**Kyushu**	14.0500**	12.2400**	8.5920**		**Kyushu**	11.6200**	8.9650**	7.7100**		Kyushu	10.1200**	9.6590**	5.4170**	

S2.2 Jomon analyses

S2.2 Jomon analyses - current ecological variables

Jomon craniofacial analysis 2-B PLS with current ecological variables

No significant covariation between blocks (RV coefficient: 0.17, P-value: 0.55). PLS1 and 2 together account for >99% covariation, so only these investigated.

Table S2.2.1: Singular values and pairwise correlations of PLS scores between blocks for current climate, craniofacial landmark set analysis:

	Singular value	P-value (perm.)	% total covar.	Correlation	P-value (perm.)
PLS1	3.07	0.63	58.27	0.74	0.6
PLS2	2.58	0.4	41.09	0.74	0.46

Table S2.2.2: Loadings of ecological variables on block 2 for current climate, craniofacial landmark set analysis.

	PLS1	PLS2	PLS3	PLS4	PLS5	PLS6	PLS7
Altitude	-0.0346	-0.9992	0.0073	-0.0113	0.0136	-0.0007	0.0003
MeanTemp	0.0085	0.0051	0.1081	0.1827	0.4755	0.1434	0.8415
MaxTemp	0.0060	0.0040	0.1102	0.1804	0.4359	0.5290	-0.3899
MinTemp	0.0095	0.0073	0.1112	0.1809	0.6438	-0.2604	-0.3731
TempRange	-0.0036	-0.0032	-0.0010	-0.0005	-0.2079	0.7894	-0.0168
AnnPrecip	0.9794	-0.0362	-0.1813	0.0813	-0.0054	0.0026	-0.0014
MaxPrecip	0.1984	0.0032	0.9009	-0.3817	-0.0569	-0.0132	-0.0005
MinPrecip	0.0100	0.0116	-0.3454	-0.8654	0.3504	0.0918	0.0185

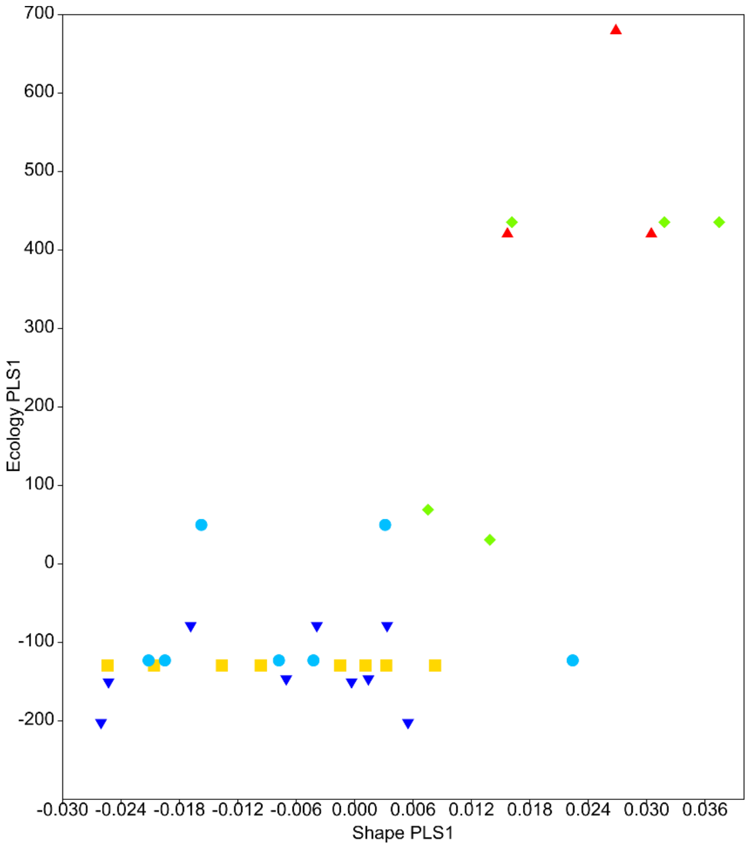

Figure S2.2.1: Block 1 PLS1 against block 2 PLS1 for Jomon craniofacial landmark set and recent climate data (no significant association). Dark blue inverted triangles: Hokkaido, light blue circles: North Honshu, green diamonds, mid Honshu, yellow squares: South Honshu, red triangles: Kyushu.

Jomon facial analysis 2-B PLS with current ecological variables

No overall association between blocks (RV coefficient: 0.17, P-value: 0.47). PLS1 and 2 together account for >99% covariation, so only these investigated.

Table S2.2.3: Singular values and pairwise correlations of PLS scores between blocks for current climate, facial landmark set:

	Singular value	P-value (perm.)	% total covar.	Correlation	P-value (perm.)
PLS1	4.29	0.22	73.89	0.71	0.66
PLS2	2.51	0.82	25.41	0.76	0.07

Table S2.2.4: Loadings of ecological variables on block 2 for current climate, facial landmark set analysis.

	PLS1	PLS2	PLS3	PLS4	PLS5	PLS6	PLS7
Altitude	0.2288	-0.9725	0.0360	-0.0155	0.0159	-0.0026	0.0005
MeanTemp	-0.0086	0.0060	0.0938	0.2001	0.4740	0.1398	0.8407
MaxTemp	-0.0058	0.0049	0.1059	0.2030	0.4386	0.5168	-0.3934
MinTemp	-0.0102	0.0087	0.0870	0.1943	0.6391	-0.2741	-0.3709
TempRange	0.0044	-0.0038	0.0189	0.0087	-0.2005	0.7909	-0.0225
AnnPrecip	-0.9569	-0.2323	-0.1607	0.0661	-0.0091	0.0050	-0.0016
MaxPrecip	-0.1772	-0.0028	0.9280	-0.3236	-0.0427	-0.0285	0.0005
MinPrecip	-0.0163	0.0051	-0.2894	-0.8784	0.3635	0.1093	0.0180

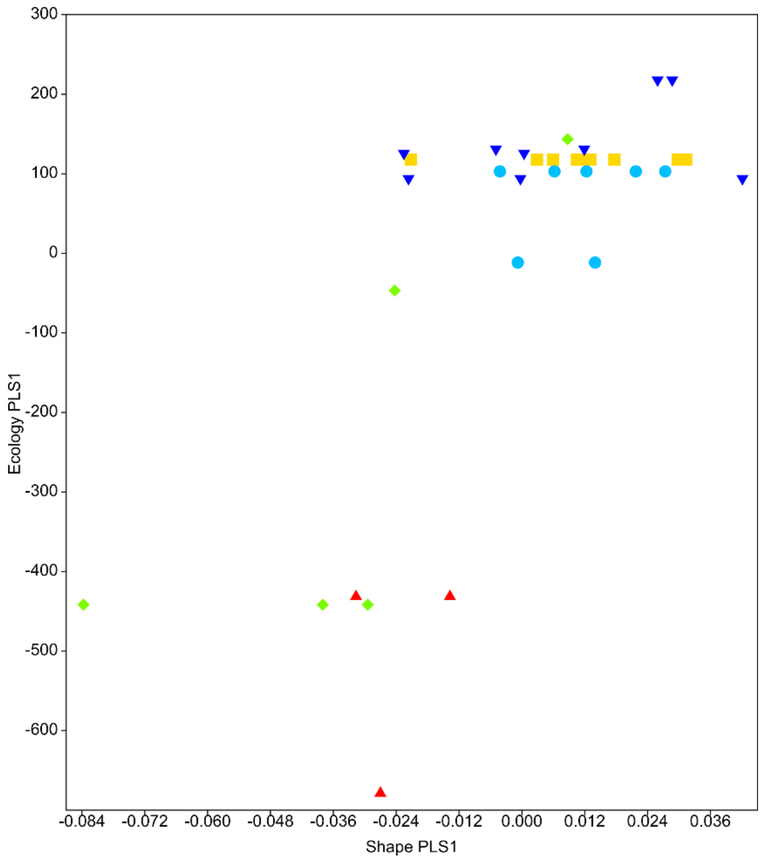

Figure S2.2.2: Block 1 PLS1 against block 2 PLS1 for Jomon facial landmark set and recent climate data (no significant association). Dark blue inverted triangles: Hokkaido, light blue circles: North Honshu, green diamonds, mid Honshu, yellow squares: South Honshu, red triangles: Kyushu.

Jomon neurocranial analyses 2-B PLS with current ecological variables

No significant covariation between blocks (RV coefficient: 0.03, P-value: 0.97). PLS1 and 2 together account for >99% covariation, so only these investigated.

Table S2.2.5: Singular values and pairwise correlations of PLS scores between blocks for current climate, neurocranial landmark set:

	Singular value	P-value (perm.)	% total covar.	Correlation	P-value (perm.)
PLS1	1.4	0.98	61.26	0.41	0.29
PLS2	1.11	0.82	37.99	0.26	0.83

Table S2.2.6: Loadings of ecological variables on block 2 for current climate, neurocranial landmark set analysis.

	PLS1	PLS2	PLS3	PLS4	PLS5	PLS6	PLS7
Altitude	0.568944	-0.82232	0.006476	-0.00573	0.004598	-0.00074	3.33E-05
MeanTemp	-0.00033	0.004212	0.162393	-0.54278	-0.08712	0.186466	0.797894
MaxTemp	-0.00167	0.001372	0.158729	-0.5241	-0.42662	-0.02756	-0.42898
MinTemp	-0.00081	0.006974	0.174198	-0.57866	0.385909	0.01394	-0.39026
TempRange	-0.00086	-0.0056	-0.01547	0.054563	-0.81252	-0.0415	-0.03872
AnnPrecip	0.811893	0.560776	-0.15631	-0.04361	-0.00469	0.001618	-0.00136
MaxPrecip	0.130875	0.09601	0.944804	0.28459	0.00038	-0.0025	0.001478
MinPrecip	-0.00058	-0.0024	-0.02687	0.099759	-0.03527	0.981093	-0.15979

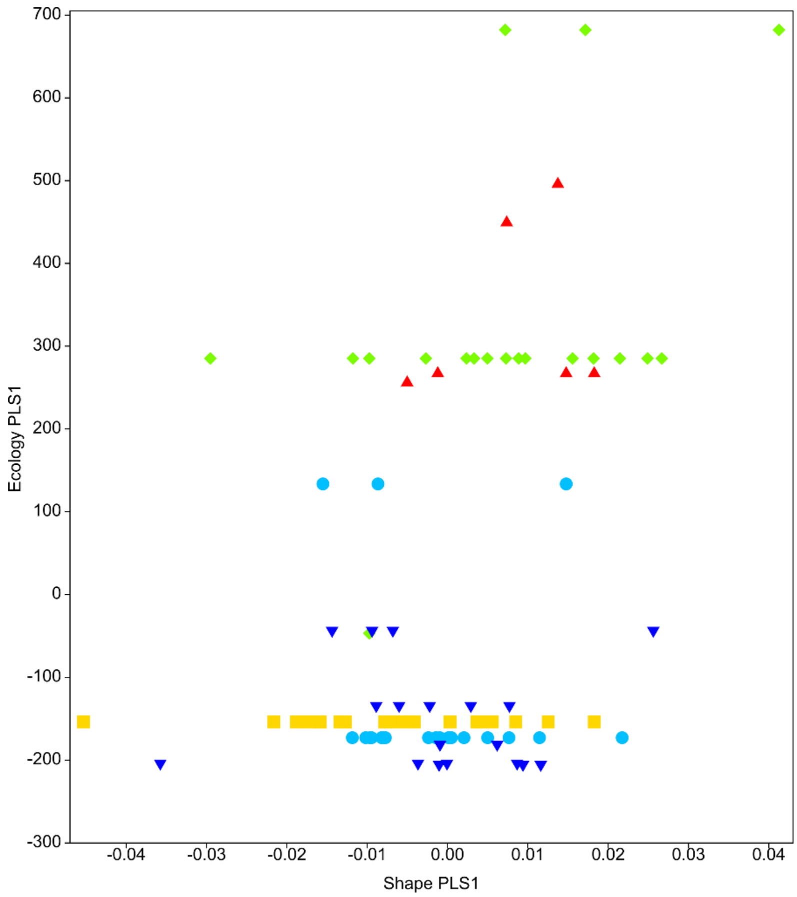

Figure S2.2.3: Block 1 PLS1 against block 2 PLS1 for Jomon neurocranial landmark set and recent climate data (no significant association). Dark blue inverted triangles: Hokkaido, light blue circles: North Honshu, green diamonds, mid Honshu, yellow squares: South Honshu, red triangles: Kyushu.

S2.3. Jomon Palaeoclimate analysis

We used additional mid-Holocene palaeoclimatic estimates for the Jomon as alternative climatic data due to the potential mis-match between modern climates for sites and conditions experienced by their Jomon inhabitants, resulting from climate change over the Holocene. All of the Jomon sites in the current sample have chronologies where at least part of the estimated date range (see Table S2.3.1) would be within the mid-Holocene period, ~8,200-3,300 BP, which was approximately 1-2°C warmer than present in Japan [1]. The WorldClim Mid-Holocene climate estimation is from ~6,000 BP [2]. The altitude values are not included in the palaeoclimatic analyses as they remained unchanged from those used in the current climate analyses.

Table S2.3.1: Details of date and housing institution for Jomon sample. SMU: Sapporo Medical University, NMNS: National Museum of Nature and Science (Tsukuba), KU: Kyoto University, FK: Kyushu University (Fukuoka). For references see section S3. In some cases the original reference is in Japanese, in these cases the reference cited is not the original reference, but the English language article in which the original reference was cited.

Specimen	Period	Date (years BP)	Institution
Funadomari	Late Jomon	3800-3500[3]	SMU
Irie	Late Jomon	4050-3000[4]	SMU
Kitakogane	Early Jomon	6100-4800[5]	SMU
Kotan-Onsen	Middle Jomon	5100-4050[4]	SMU
Takasago	Final Jomon	3000-2500[6]	SMU
Ebishima	Middle-Final Jomon	5000-2300[7]	NMNS
Miyano	Middle-Final Jomon	5000-2300[7]	NMNS
Ikawazu	Middle-Final Jomon	5000-2300[8]	NMNS
Tochibara	Middle-Final Jomon	5000-2300[8]	NMNS
Wakaumi	Middle Jomon	5000-3000[7]	NMNS
Yoshigo	Late-Final Jomon	4000-2500[9]	KU
Tsukumo	Late-Final Jomon	4000-2500[9]	KU
Einomaru	Final Jomon	3500-2300[10]	FK
Goryo	Final Jomon	3500-2300[10]	FK
Todoroki	Early-Middle Jomon	5500-3000[11]	KU
Yamaga	Final Jomon	3500-2300[10]	FK

Jomon craniofacial palaeoclimatic 2-B PLS analyses

There is no overall association between blocks (RV coefficient: 0.15, P-value: 0.24). PLS1 accounts for >90% covariation, so this is the only PLS investigated.

Table S2.3.2: Singular values and pairwise correlations of PLS scores between blocks for palaeoclimate, craniofacial landmark set:

	Singular value	P-value (perm.)	% total covar.	Correlation	P-value (perm.)
PLS1	0.08	0.25	93.81	0.77	0.29

Table S2.3.3: Loadings of ecological variables on block 2 for palaeoclimate, craniofacial landmark set analysis.

	PLS1	PLS2	PLS3	PLS4	PLS5	PLS6	PLS7
**MeanTemp**	0.5687	-0.0276	0.1192	-0.1736	0.1553	0.2008	-0.7530
**MaxTemp**	0.5188	0.3507	0.6375	0.1037	-0.0886	-0.1696	0.3925
**MinTemp**	0.6164	-0.2314	-0.5987	0.2131	-0.1611	0.1515	0.3373
**TempRange**	-0.1281	0.2034	0.1298	-0.0995	-0.3888	0.8695	0.0909
**AnnPrecip**	0.0547	-0.4486	0.1907	-0.4505	0.5879	0.2560	0.3812
**MaxPrecip**	0.0556	-0.3634	0.0882	-0.5991	-0.6485	-0.2784	-0.0005
**MinPrecip**	-0.0705	-0.6694	0.3998	0.5847	-0.1572	0.0944	-0.1075

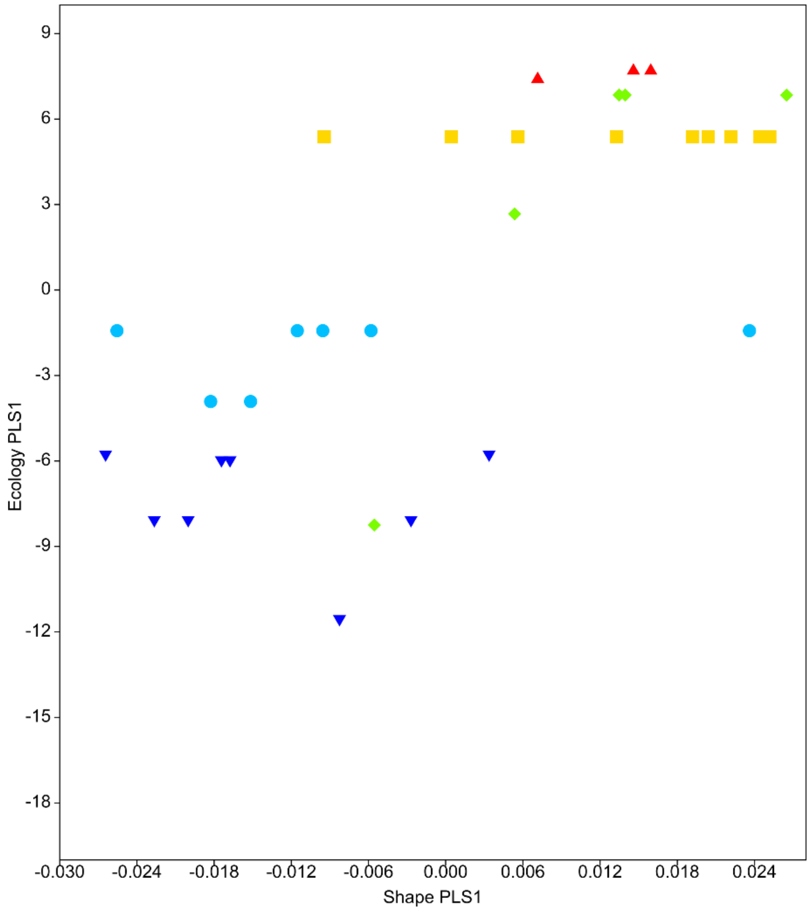

Figure S2.3.1: Block 1 PLS1 against block 2 PLS1 for Jomon craniofacial landmark set and palaeoclimatic data (no significant association). Dark blue inverted triangles: Hokkaido, light blue circles: nNrth Honshu, green diamonds, mid Honshu, yellow squares: South Honshu, red triangles: Kyushu.

Jomon facial palaeoclimatic 2-B PLS analyses

There is no overall association between blocks (RV coefficient: 0.21, P-value: 0.09). PLS1 and PLS2 account for >95% covariation, so these are the only two PLS factors investigated.

Table S2.3.4: Singular values and pairwise correlations of PLS scores between blocks for palaeoclimate, facial landmark set:

	Singular value	P-value (perm.)	% total covar.	Correlation	P-value (perm.)
PLS1	0.04	0.15	67.57	0.72	0.39
PLS2	0.02	0.07	28.09	0.73	0.18

Table S2.3.5: Loadings of ecological variables on block 2 for palaeoclimate, facial landmark set analysis.

	PLS1	PLS2	PLS3	PLS4	PLS5	PLS6	PLS7
MeanTemp	0.4813	-0.1033	0.0419	-0.0417	0.1271	-0.8590	0.0089
MaxTemp	0.4857	-0.2436	0.0692	-0.2750	0.4914	0.3957	0.4758
MinTemp	0.4796	-0.0700	0.1945	-0.0469	-0.0085	0.2793	-0.8043
TempRange	-0.4245	-0.1647	-0.3377	-0.2579	0.6857	-0.1242	-0.3558
AnnPrecip	0.2403	0.4725	-0.3528	0.6776	0.3588	0.0809	0.0016
MaxPrecip	0.2387	0.3473	-0.6752	-0.5276	-0.2941	0.0411	-0.0031
MinPrecip	-0.0870	0.7444	0.5110	-0.3412	0.2387	-0.0614	0.0029

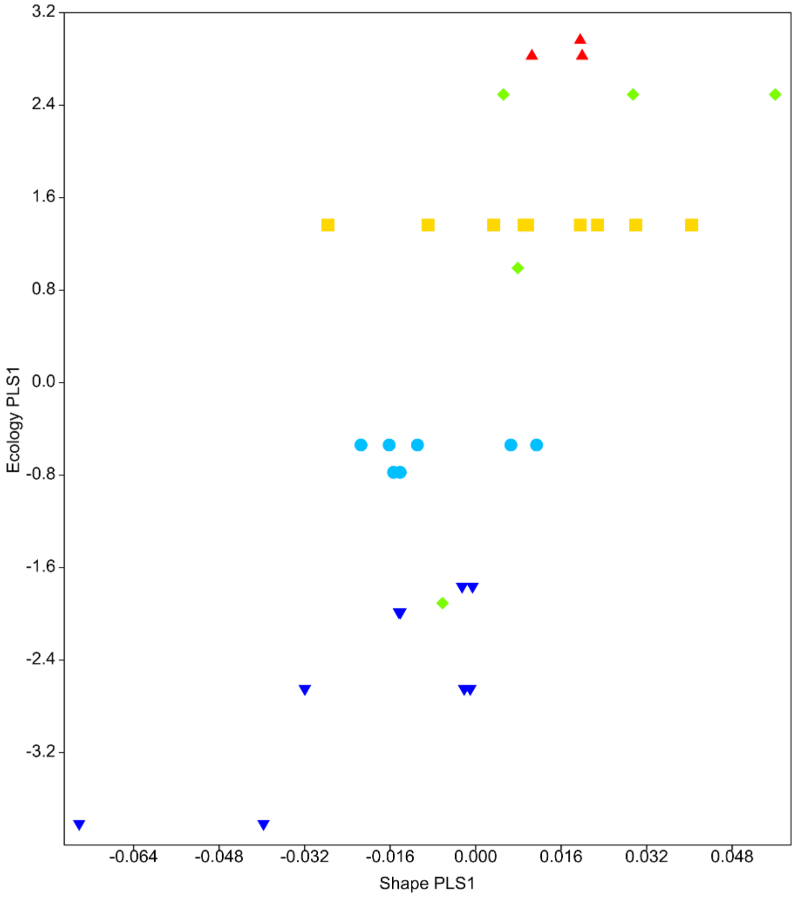

Figure S2.3.2: Block 1 PLS1 against block 2 PLS1 for Jomon facial landmark set and palaeoclimatic data (no significant association). Dark blue inverted triangles: Hokkaido, light blue circles: North Honshu, green diamonds, mid Honshu, yellow squares: South Honshu, red triangles: Kyushu.

Jomon neurocranial palaeoclimatic 2-B PLS analyses

There is no overall association between blocks (RV coefficient: 0.07, P-value: 0.14). PLS1 and PLS2 account for >95% covariation, so these are the only two PLS factors investigated.

Table S2.3.6: Singular values and pairwise correlations of PLS scores between blocks for palaeoclimate, neurocranial landmark set:

	Singular value	P-value (perm.)	% total covar.	Correlation	P-value (perm.)
PLS1	0.02	0.12	80.32	0.49	0.01
PLS2	0.01	0.51	15.78	0.31	0.64

Table S2.3.7: Loadings of ecological variables on block 2 for palaeoclimate, neurocranial landmark set analysis.

	PLS1	PLS2	PLS3	PLS4	PLS5	PLS6	PLS7
MeanTemp	0.4185	-0.2314	0.0823	-0.1640	0.0794	-0.8551	-0.0136
MaxTemp	0.4658	-0.1389	0.2039	-0.4799	0.3094	0.4135	-0.4745
MinTemp	0.3846	-0.2739	0.2300	-0.0092	0.0021	0.2737	0.8057
TempRange	-0.2399	0.4231	-0.2430	-0.6209	0.4278	-0.1021	0.3542
AnnPrecip	-0.0638	-0.4917	-0.5170	0.3102	0.6230	0.0505	-0.0044
MaxPrecip	0.0432	-0.3449	-0.6089	-0.4183	-0.5715	0.0829	0.0044
MinPrecip	-0.6297	-0.5589	0.4492	-0.2932	0.0177	-0.0558	-0.0021

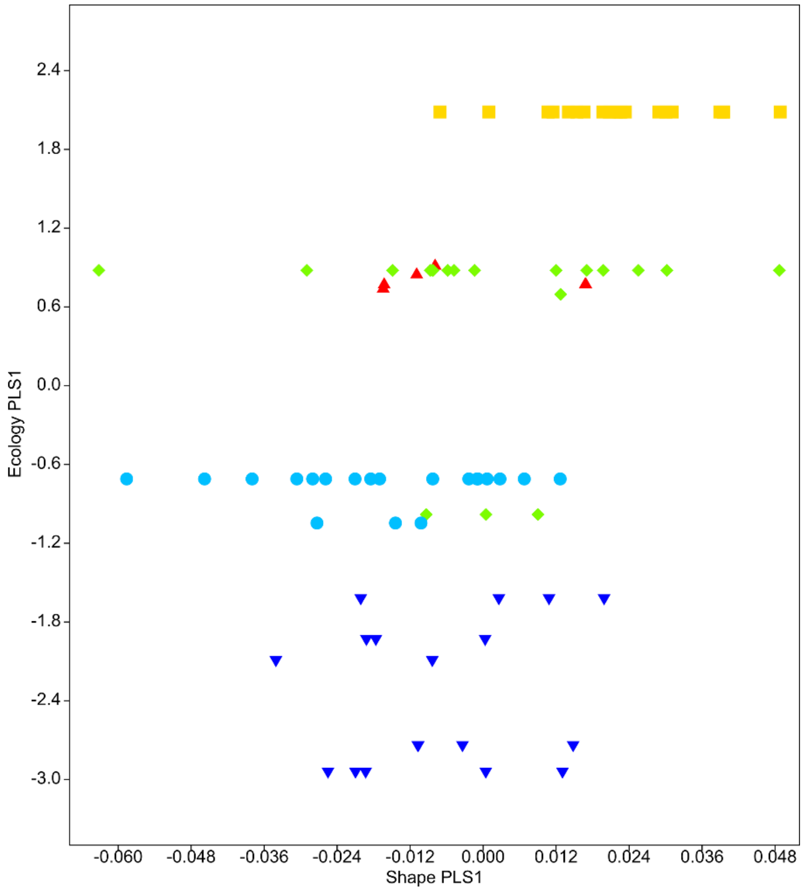

Figure S2.3.3: Block 1 PLS1 against block 2 PLS1 for Jomon neurocranial landmark set and palaeoclimatic data (no significant association). Dark blue inverted triangles: Hokkaido, light blue circles: North Honshu, green diamonds, mid Honshu, yellow squares: South Honshu, red triangles: Kyushu.

As with the current climate analyses, there is no significant relationships between Jomon craniofacial, facial, or neurocranial morphology and palaeoclimatic variables. This supports the suggestion that ecology is not a key driver of Jomon craniofacial morphology.

S.3 References

1. Koizumi, I. Diatom-derived SSTs (Td' ratio) indicate warm seas off Japan during the middle Holocene (8.2-3.3 kyr BP). *Mar. Micropaleontol.* **69,** 263–281 (2008).
2. Hijmans, R. J., Cameron, S. E., Parra, J. L., Jones, P. G. & Jarvis, A. Very high resolution interpolated climate surfaces for global land areas. *Int. J. Climatol.* **25,** 1965–1978 (2005).
3. Adachi, N., Shinoda, K. I., Umetsu, K. & Matsumura, H. Mitochondrial DNA analysis of jomon skeletons from the funadomari site, hokkaido, and Its implication for the origins of native american. *Am. J. Phys. Anthropol.* **138,** 255–265 (2009).
4. Oxenham, M. F. & Matsumura, H. Oral and Physiological Paleohealth in Cold Adapted Peoples: Northeast Asia, Hokkaido. *Am. J. Phys. Anthropol.* **135,** 64–74 (2008).
5. Yoneda, M. *et al.* Radiocarbon marine reservoir effect in human remains from the Kitakogane site, Hokkaido, Japan. *J. Archaeol. Sci.* **29,** 529–536 (2002).
6. Adachi, N. *et al.* Mitochondrial DNA analysis of Hokkaido Jomon skeletons: Remnants of archaic maternal lineages at the southwestern edge of former Beringia. *Am. J. Phys. Anthropol.* **146,** 346–360 (2011).
7. Hagihara, Y. & Nara, T. Morphological features of the fibula in Jomon hunter-gatherers from the shell mounds of the Pacific coastal area. *Am. J. Phys. Anthropol.* **160,** 708–718 (2016).
8. Matsumura, H. Non-metric dental trait variation among local sites and regional groups of the Neolithic Jomon period, Japan. *Anthropol. Sci.* **115,** 25–33 (2007).
9. Temple, D. H., Auerbach, B. M., Nakatsukasa, M., Sciulli, P. W. & Larsen, C. S. Variation in limb proportions between Jomon foragers and Yayoi agriculturalists from prehistoric Japan. *Am. J. Phys. Anthropol.* **137,** 164–174 (2008).
10. Nakahashi, T. Temporal craniometric changes from the Jomon to the Modern period in western Japan. *Am. J. Phys. Anthropol.* **409425,** (1993).
11. Takayama, J. Incised Human Figures from Mesolithic Japan. *Arctic Anthropol.* **5,** 68–71 (1968).
